# Immunomodulatory Effects of Tokishakuyakusan on Progesterone Withdrawal–Induced Uterine Inflammation in Pregnant Mice

**DOI:** 10.1111/aji.70318

**Published:** 2026-09-04

**Authors:** Takeshi Nagamatsu, Manami Yanagisawa, Yohei Tokita, Keisuke Nakajima, Tomohiro Otani, Ayumi Taguchi, Mari Ichinose, Keiich Kumasawa, Takayuki Iriyama

**Affiliations:** ^1^ Department of Obstetrics, Gynecology International University of Health and Welfare Narita Chiba Japan; ^2^ Department of Obstetrics and Gynecology, Faculty of Medicine The University of Tokyo Tokyo Japan; ^3^ TSUMURA Kampo Research Laboratories, Kampo Research and Development Division TSUMURA & CO. Tokyo Japan

**Keywords:** inflammation, progesterone, pregnancy, Tokishakuyakusan

## Abstract

**Problem:**

Tokishakuyakusan (TSS) is a traditional Japanese Kampo medicine widely used to support pregnancy; however, its molecular mechanisms remain poorly understood. This study aimed to elucidate the progesterone‐related immunomodulatory mechanisms underlying the pregnancy‐supportive effects of TSS using a mouse model of progesterone withdrawal induced by mifepristone.

**Methods:**

Pregnant mice were fed either a control diet or a diet containing 1% TSS from mating until late gestation. On day 15 postcoitus, all mice received low‐dose mifepristone to induce functional progesterone withdrawal. Cytokine and chemokine profiles in serum, corpus uteri, and placenta were analyzed using multiplex assays, and uterine expression of progesterone‐responsive and inflammatory genes were evaluated by real‐time RT‐PCR.

**Results:**

Mifepristone administration induced a systemic proinflammatory response, characterized by increased serum levels of G‐CSF, IL‐6, KC, and MCP‐1. TSS administration increased G‐CSF without affecting fetal number, fetal weight, or placental weight. In the corpus uteri, TSS significantly suppressed IL‐6 protein expression, whereas placental cytokine profiles were largely unaffected. Furthermore, TSS significantly increased uterine mRNA expression of the progesterone‐responsive anti‐inflammatory molecules secretory leukocyte peptidase inhibitor (SLPI) and progranulin (PGRN).

**Conclusions:**

These findings suggest that TSS mitigates inflammation associated with progesterone withdrawal by suppressing uterine IL‐6 and increasing the expression of progesterone‐responsive anti‐inflammatory molecules such as SLPI and PGRN. These findings suggest that TSS may modulate progesterone‐responsive anti‐inflammatory pathways through increased expression of SLPI and PGRN while suppressing uterine IL‐6.

## 1 Introduction

Tokishakuyakusan (TSS) is a traditional Japanese medicine (kampo) widely prescribed to support pregnancy. It is commonly used to treat pregnant women with conditions such as recurrent pregnancy loss (RPL), threatened preterm labor, and fetal growth restriction. Under the Japanese healthcare system, TSS is covered by national insurance, reflecting its integration into conventional obstetric care. In previous report, a propensity score matched study demonstrated that TSS significantly increases the live birth rate over two‐years among women suffering from RPL [1]. Furthermore, a national cohort study reported that approximately 1.0% of the pregnant women in Japan were prescribed TSS during pregnancy [2]. Despite its widespread use, the molecular mechanisms underlying the therapeutic effects of TSS remain largely unexplored.

In previous studies on the molecular mechanisms of TSS, accumulating evidence from in vitro and in vivo studies suggested that the pharmacological effects of TSS are associated with anti‐inflammatory actions and the modification of sex hormone signaling [3, 4]. In the research context of endometriosis, TSS have been demonstrated to suppress proinflammatory cytokines, including IL‐33 and IL‐8, consequently leading to prevention of disease progression [5]. In a rat model of implantation failure, TSS reversed the mifepristone‐induced downregulation of leukemia inhibitory factor (LIF) and interleukin ‐11 (IL‐11) expression, demonstrating its potential to enhance implantation [4]. Our previous research further demonstrated that the TSS mitigates miscarriage by inhibiting the activation of invariant natural killer T (iNKT) cell [3].

The pathogenesis of preterm birth and the role of progesterone are closely related. Progesterone plays a pivotal role in maintaining pregnancy by modulating immune tolerance and uterine quiescence [6]. A decline in progesterone levels is associated with abnormality in pregnancy outcomes, including miscarriage or premature birth [7, 8]. During human gestation, progesterone is initially produced by the corpus luteum and later by the placenta from mid‐pregnancy period onward. Mifepristone, a progesterone receptor antagonist, induces miscarriage when administered during early pregnancy and triggers labor in late gestation [9]. We previously reported that mifepristone‐induced preterm birth is accompanied by the downregulation of anti‐inflammatory molecules such as secretory leukocyte peptidase inhibitor (SLPI) and progranulin (PGRN) [10].

This study aimed to elucidate the mechanisms by which TSS contributes to pregnancy maintenance, with a particular focus on its relevance to progesterone. Using a mouse model of pregnancy failure induced by mifepristone, we comprehensively investigated the effects of TSS on the inflammation‐related factors regulated by progesterone.

## 2 Material and Methods

### 2.1 Drugs

TSS extract powder (lot no. 2180023010), which was manufactured as a Japanese traditional medicine approved by the Ministry of Health, Labour and Welfare for clinical use (see STORK: http://mpdb.nibiohn.go.jp/stork/), was supplied by Tsumura & Co. (Tokyo, Japan). To manufacture TSS, a mixture comprising 6 crude drugs (Peony Root, Atractylodes Lancea Rhizome, Alisma Rhizome, Poria Sclerotium, Cnidium Rhizome and Japanese Angelica Root) was extracted with purified hot water at 95°C for 1 h. The extract solution was separated from the insoluble waste and concentrated by removing water under reduced pressure. By spray‐drying it, the dried extract powder of TSS was finally obtained from the mixture. The yield of the extract was 18.2%. The study diet was prepared by mixing dry powdered extracts of TSS with standard laboratory food (CE‐2, CLEA Japan, Inc., Tokyo, Japan) at a final concentration of 1.0%.

### 2.2 The Mifepristone‐Induced Withdrawal of Progesterone in Pregnant Mice

All experiments were performed under the approval of the Institutional Animal Care and Use Committee of the University of Tokyo. C57BL/ 6J mice were purchased from Japan SLC, Inc (Hamamatsu, Shizuoka, Japan). Female mice were mated at 9 to 12 weeks of age. Day 0.5 postcoitus (pc) was defined as the morning of vaginal plug detection and mice with a vaginal plug were randomly divided into two groups at this timepoint. There were no significant differences between the two groups in terms of age, body weight, pregnancy rate and number of embryos per pregnancy. The animals were allowed free access to water and food. standard laboratory food. From day 0.5 pc until the day of the following assessment, the intervention group of mice was fed a diet containing 1% TSS (Tsumura & Co, Tokyo, Japan) and the control group were given the standard laboratory food (CE‐2, CLEA Japan, Inc., Tokyo, Japan) without TSS. On day 15 pc, mifepristone (Sigma‐Aldrich, St. Louis, MO, USA) was administrated intraperitoneally at a dose of 100 µg/body to all animals. Tail vein blood was sampled immediately at 12 and 18 h after mifepristone administration. The dams were sacrificed, and the incidence of the fetal and placental evaluation was evaluated until 18 h after mifepristone administration. Fetal and placental weights were calculated as the average values. Premature birth occurred in a few individuals until 18 h after mifepristone administration. In this study, we evaluated pregnant animals that had not caused preterm birth. The mice was sacrificed at 18 h after mifepristone administration and the uterus and placental tissues were stocked at ‐80°C for the quantitative analysis. Fetoplacental weights were determined.

### 2.3 Multiplex Cytokine Arrays

Serum sample and protein of corpus uteri and placenta were extracted and analyzed for cytokine/chemokine expression. Proteins were extracted by soaking corpus uteri and placenta in PBS containing 1% protease inhibitor cocktail and homogenized using MagNA Lyser (Roche Diagnostics K.K., Tokyo, Japan), ultra sonicating, centrifuging and collecting supernatant. The samples were stored at ‐80°C until analysis.

Cytokine levels in each samples for Granulocyte‐colony stimulating factor (G‐CSF), Granulocyte‐Macrophage Colony‐Stimulating Factor (GM‐CSF), interferon (IFN) γ, macrophage inflammatory protein 3 (MIP‐3), interleukin (IL) 1β, IL‐4, IL‐6, IL‐10, Keratinocyte‐derived chemokines/Growth‐related oncogene (KC), Monocyte Chemotactic Protein 1 (MCP‐1), Macrophage Inflammatory Protein (MIP)‐1a, MIP‐2, and Tumor Necrosis Factor (TNF)‐α were measured using a cytokine array system; Bio‐Plex Suspension Array System (Bio‐Rad Laboratories, Hercules, CA) with MILLIPLEX MAP Mouse Cytokine/Chemokine Magnetic Bead Panel (Millipore Corp., Billerica, MA) according to the manufacturer's instructions. The minimum detectable concentrations of each cytokine are as follows: 1.7 pg/ml (G‐CSF), 10.9 pg/ml (GM‐CSF), 1.1 pg/ml (IFN‐γ), 5.4 pg/ml (IL‐1β), 0.4 pg/ml (IL‐4), 1.1 pg/ml (IL‐6), 2.0 pg/ml (IL‐10), 2.3 pg/ml (KC), 6.7 pg/ml (MCP‐1), 7.7 pg/ml (MIP‐1α), 30.6 pg/ml (MIP‐2) and 2.3 pg/ml (TNF‐α).

### 2.4 Real‐Time RT‐PCR

Measurements of mRNA have been described in a previous report (PMID: 37837670). In briefly, total RNA from corpus uteri was isolated using a tissue total RNA mini kit 157 (FAVORGEN Biotech, Changzhi, China) according to manufacturer's instructions. The purity of the recovered RNA was determined by measuring the absorbance ratio (260/280 nm) using a spectrophotometer (Epoch Micro‐Volume Spectrophotometer System: BioTek, Winooski, Vermont). Only samples with high purity based on the absorbance ratio (1.7–2.1) were subjected to analysis. Total RNA was reverse‐transcribed into cDNA, and quantitative real‐time PCR was performed using a Light Cycler thermal cycler system (Roche). The expression levels were normalized to that of β‐actin. Oligonucleotide primer sequences for the real‐time RT‐PCR are shown in Table 1.

**TABLE 1:** Oligonucleotide primer sequences for real‐time RT‐PCR.

Gene symbol	Forward (5’→3’)	Reverse (5’→3’)	Accession No.
*Slpi*	CCTTTCACGGTGCTCCTT	GGCAGACTTTCCCACATATA	NM_011414.4
*Grn*	GGTTGATGGTTCGTGGGGATGTTG	AAGGCAAAGACACTGCCCTGTTGG	BC049943.1
*Il6*	AACGATGATGCACTTGCAGA	GAGCATTGGAAATTGGGGTA	M20572.1
*Tnf*	TCTTCTGTCTACTGAACTTCGGGGTGA	GTGGTTTGCTACGACGTGGGCTA	M13049.1
*Actb*	GCCTTCCTTCTTGGGTATGG	CGGATGTCAACGTCACACTT	BC138614.1

### 2.5 Statistical Analysis

All data are presented as the median [25th and 75th percentiles]. The graphs are presented as box‐and‐whisker plots. The box represents the interquartile range (25th‐75th percentiles), and the whiskers indicate the minimum and maximum values. Comparisons of the fetal outcomes and cytokine/chemokine levels between two groups were performed using the Mann‐Whitney test. A two‐group comparison in the serum cytokine/chemokine levels were performed using gatekeeping as follows. Prior to multiple comparisons, analysis of variance in each time points in the control group were assessed using Bartlett's test, we performed Dunnett's test or Steel dwass test. The comparison test between the two groups (Mann‐Whitney test) was performed only on the points at which the control group showed a statistically significant difference from 0 h. *P* < 0.05 was considered statistically significant. All the statistical analyses were performed using GraphPad Prism (version 10.6.0, GraphPad Software, San Diego, CA, USA) and StatLight2000 (Yukms Co., Ltd, Kanagawa, Japan).

## 3 Results

### 3.1 Effects of Fetal Outcomes

**FIGURE 1:**
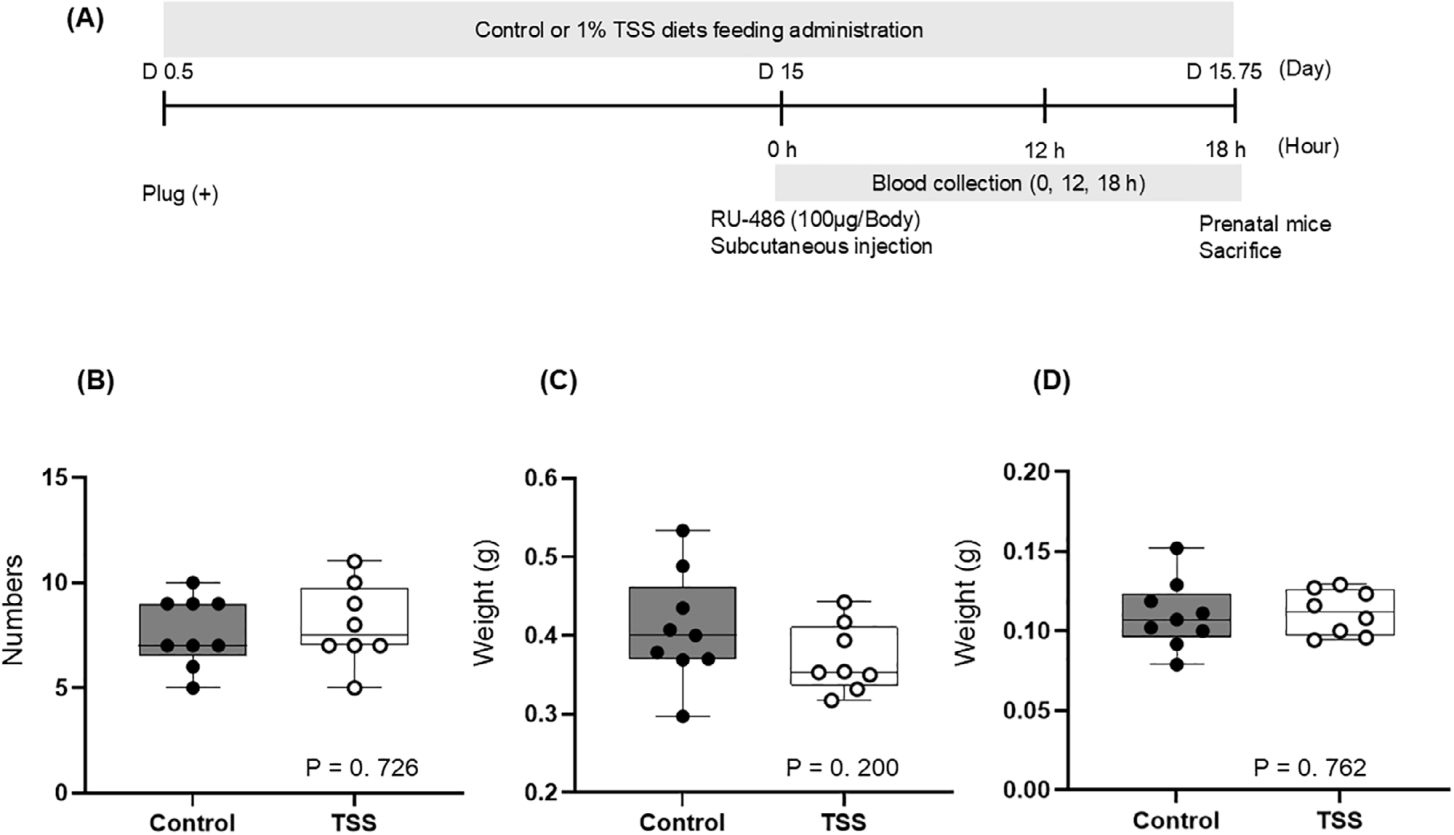
Experimental design and effect of fetal outcomes. (A) Female mice were mated, and mice with vaginal plugs were randomly allocated to the control and TSS group. 1%TSS was administered until assessment. mifepristone (100 µg/body) was administrated intraperitoneally to all animals. Each group was evaluated at the 0, 12 and 18 h after mifepristone administration. (B) The number of fetus, (C) The weight of fetus and (D) The placenta weight. The number and weight of fetus, and placenta weight were not affected. Each value represents the median (25th and 75th percentiles, *n* = 8 and 9). Statistical significance was determined by Mann‐Whitney test.

Figure 1 shows experimental design and effect of fetal outcomes. (A) Mice were administered 1% TSS from 0.5 pc until the day assessment. On the 15 days, all mice were administrated mifepristone (100 µg/body) intraperitoneally. Mice were evaluated at 0, 12, and 18 h after mifepristone administration. The number of fetus in control group was 7.0 [6.5, 9.0], and 7.5 [7.0, 9.8] in the 1%TSS group (*p* = 0.726, Figure 1B). The weight of fetus was also not significantly different between the control group (0.40 [0.37, 0.46] g) and 1%TSS group (0.35 [0.34, 0.41] g (*p* = 0.200, Figure 1C). Further, there was no significant difference in placenta weight between the control group (0.11 [0.10, 0.12] g) and 1%TSS group (0.11 [0.10, 0.13] g, *p* = 0.762, Figure 1D).

### 3.2 The Cytokines/Chemokines Profiling in the Serum

Peripheral levels of cytokines and chemokines were summarized in Table 2. Elevation of the serum levels after mifepristone administration was observed in G‐CSF, IL6, KC and MCP‐1, while the change in the serum concentration was not remarkable in the other cytokines and chemokines. On the other hand, TSS treatment was observed to enhance G‐CSF at 12 h after mifepristone administration, though no significant differences were observed in other cytokines/chemokines.

**TABLE 2:** Serum cytokine/chemokine profiling.

	Control			TSS		
	0	12	18	0	12	18
G‐CSF	639.3 [502.5, 747.0]	2613.6 [1494.7, 6638.6]^b^	2262.1 [1150.8, 5709.3]^b^	550.3 [499.5, 625.2]	8300.8 [4514.2, 41852.0]^c^	3480.9 [1353.1, 14319.1]
GM‐CSF	6.7 [6.7, 6.7]	6.7 [6.7, 6.7]	6.7 [6.7, 14.0]	6.7 [6.7, 6.7]	6.7 [6.7, 6.7]	6.7 [6.7, 42.1]
IFN‐g	1.6 [1.5, 2.3]	1.7 [1.4, 2.8]	1.6 [1.3, 1.9]	1.6 [1.5, 3.1]	1.6 [1.6, 2.2]	4.3 [1.6, 5.1]
IL‐1b	1.6 [1.4, 2.4]	2.3 [1.5, 4.0]	2.2 [1.4, 2.4]	1.6 [1.5, 1.6]	2.2 [1.3, 3.1]	2.2 [1.6, 3.4]
IL‐4	1.2 [0.8, 1.6]	1.1 [0.9, 1.7]	1.2 [0.9, 1.4]	1.1 [0.7, 1.7]	0.4 [0.3, 1.1]	1.4 [0.5, 1.6]
IL‐6	6.4 [2.7, 13.9]	486.0 [260.8, 2739.5]^b^	49.3 [17.5, 5979.8]^a^	3.2 [2.7, 5.2]	1316.1 [501.8, 7721.8]	41.1 [27.2, 1514.8]
IL‐10	5.9 [3.6, 10.0]	8.3 [6.5, 103.4]	7.1 [4.8, 353.8]	1.5 [1.5, 2.3]	7.7 [2.9, 73.7]	7.1 [2.5, 8.8]
KC	139.5 [124.8, 170.9]	894.3 [501.0, 1915.7]^a^	394.8 [225.7, 1564.5]^a^	114.0 [94.9, 122.4]	1526.2 [755.8, 3015.8]	335.5 [308.5, 1319.1]
MCP‐1	24.0 [10.0, 52.9]	98.2 [43.6, 307.2]	84.4 [47.0, 756.8]^a^	6.7 [1.3, 15.2]	140.5 [24.8, 549.0]	66.3 [37.7, 187.9]
MIP‐1a	109.8 [97.5, 164.2]	83.6 [74.1, 121.3]	97.5 [82.0, 122.8]	95.7 [66.1, 151.2]	100.0 [55.4, 128.4]	84.2 [59.2, 116.5]
MIP‐2	205.5 [176.6, 224.7]	176.6 [164.2, 196.8]	179.4 [113.4, 253.7]	125.9 [54.1, 164.2]	168.4 [82.5, 191.6]	151.2 [94.1, 176.6]
TNF‐a	9.8 [6.7, 11.5]	10.5 [7.2, 16.2]	11.2 [8.6, 21.1]	7.3 [6.2, 8.1]	10.3 [7.1, 14.5]	10.4 [7.5, 12.1]

^a^ Data are presented as the median (25th and 75th percentiles). *p*‐value obtained by Bartlett's test with steel dwass test.

^b^ *p* < 0.05 and 0.01, control: *n* = 9–11).

^c^ *P* < 0.05, versus control group at each point (Mann‐Whitney test).

### 3.3 Effects of TSS on the Cytokine/Chemokine Levels in Corpus Uteri and Placenta

We investigated the effects of TSS on cytokine/chemokine profiles in the mifepristone‐induced pregnancy abnormality model. The cytokines in the corpus uteri and placenta were measured at18 h after the mifepristone.

**FIGURE 2:**
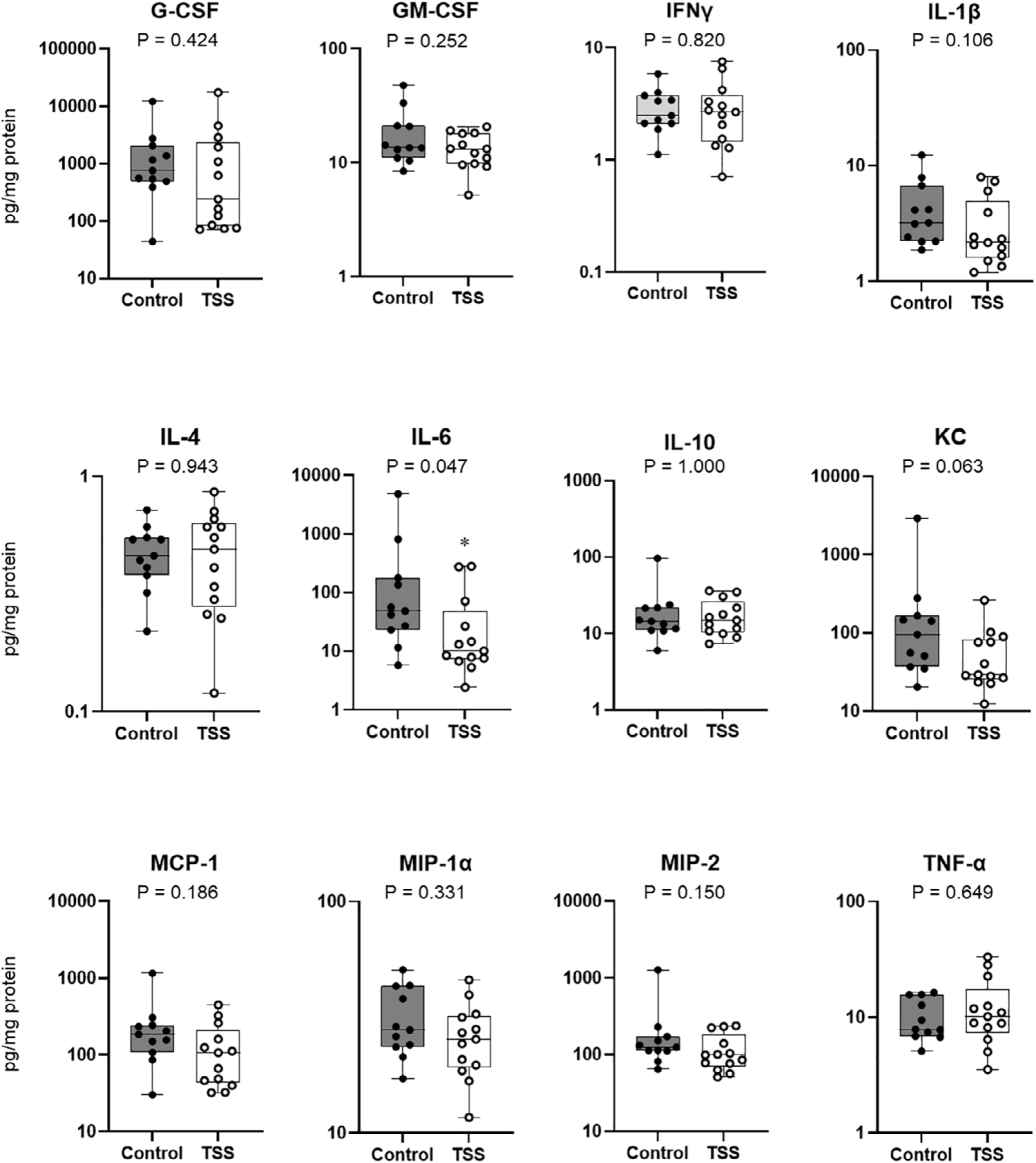
Cytokine and chemokine profiling in the corpus uter. The corpus uteri were analyzed for cytokine and chemokine levels using the Bio‐Plex Suspension Array System (Bio‐Rad Laboratories). Each value represents the median (25th and 75th percentiles, *n* = 11–13). Statistical significance was determined by Mann‐Whitney test. IL‐6 was significantly lower in the TSS group compared to the control group (*p* = 0.047).

In the analysis of protein levels in the corpus uteri (Figure 2), IL‐6 levels in the 1% TSS showed value at 10.1 [7.2, 49.1] pg/mg protein (*n* = 13) and which was suppressed compared to the control (48.6 [23.1, 179.1] pg/mg protein, *n* = 11, *p* = 0.047). KC levels were lower in the 1% TSS group (29.3 [25.1, 82.8] pg/mg protein, *n* = 13) than in the control group (95.4 [36.8, 165.6] pg/mg protein, *n* = 11), although the difference was not statistically significant (*p* = 0.063). Il‐1β, MIP‐2, and MCP‐1 levels were not significantly affected between the control group (3.2 [2.2, 6.8], 124.6 [111.7, 171.9], and 185.3 [107.5, 243.3] pg/mg protein, respectively. each analyte, *n* = 11) and 1%TSS group (2.2 [1.6, 5.0], 99.0 [69.9, 182.6], and 107.1 [43.3, 211.1] pg/mg protein respectively, showing a tendency for suppression with 1% TSS administration (*p* = 0.106, 0.150 and 0.186 respectively, *n* = 13). While no changes were observed in Th2 cytokines, IL‐4 and IL‐10. Further the other cytokines were not significantly affected. The 1% TSS group showed a higher IL‐10 level (0.6 [0.5, 3.3] pg/mg protein) than the control group (0.5 [0.2, 0.6] pg/mg protein), although the difference was not statistically significant (*p* = 0.134). There were no differences in other cytokines between the 1% TSS and control groups.

**FIGURE 3:**
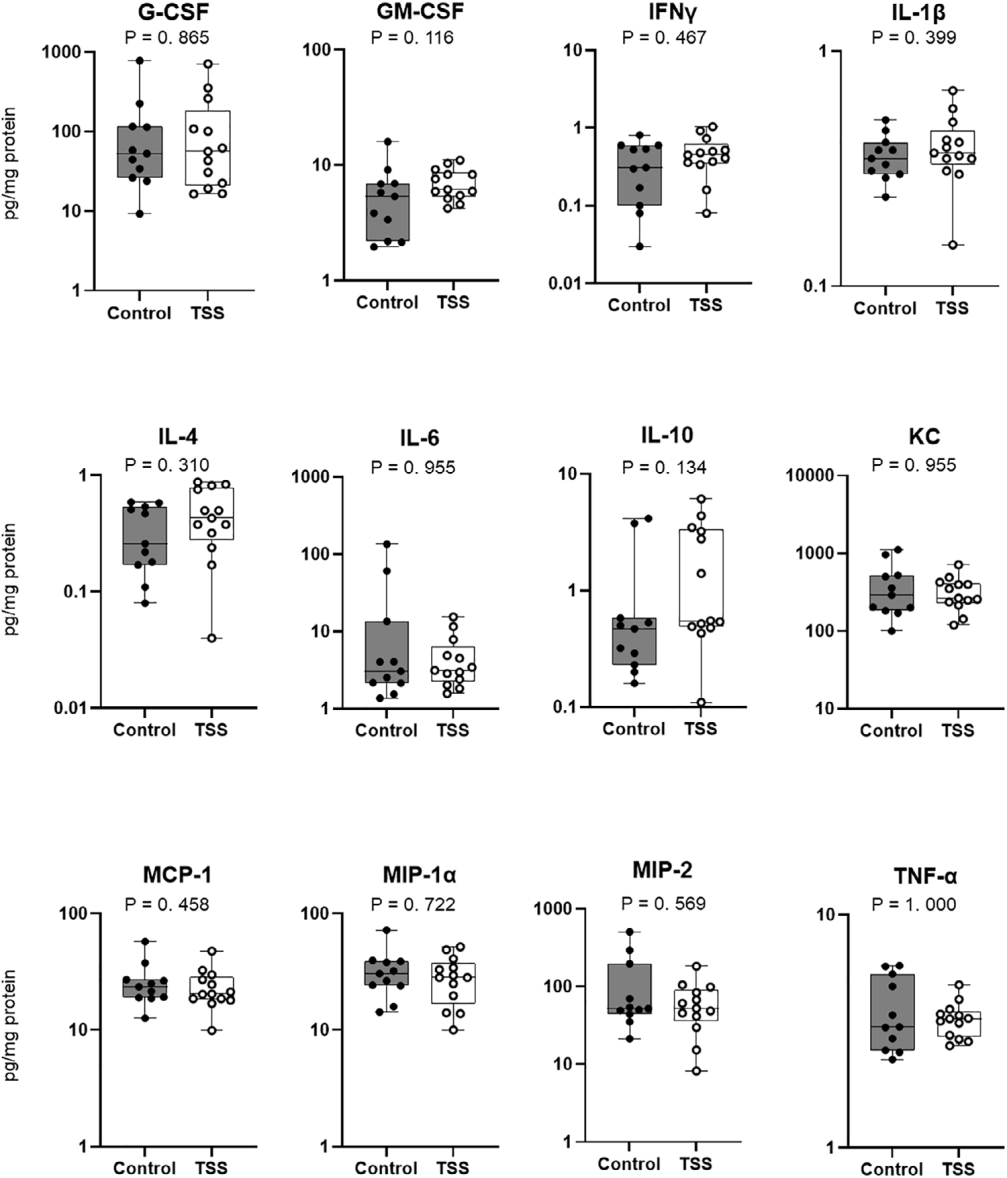
Cytokine and chemokine profiling in the placenta. The placenta were analyzed for cytokine and chemokine levels using the Bio‐Plex Suspension Array System (Bio‐Rad Laboratories). Each value represents the median (25th and 75th percentiles, *n* = 11–13). Statistical significance was determined by Mann‐Whitney test. TSS administration did not significantly affect the placental levels of any of the cytokines or chemokines examined.

In contrast to the findings in the placenta, TSS administration did not significantly affect the placental levels of any of the cytokines or chemokines examined, including G‐CSF, GM‐CSF, IFN‐γ, IL‐1β, IL‐4, IL‐6, IL‐10, KC, MCP‐1, MIP‐1α, MIP‐2, and TNF‐α (all *P* ≥ 0.116; Figure 3).

### 3.4 Effects of TSS on the Gene Expression

**FIGURE 4:**
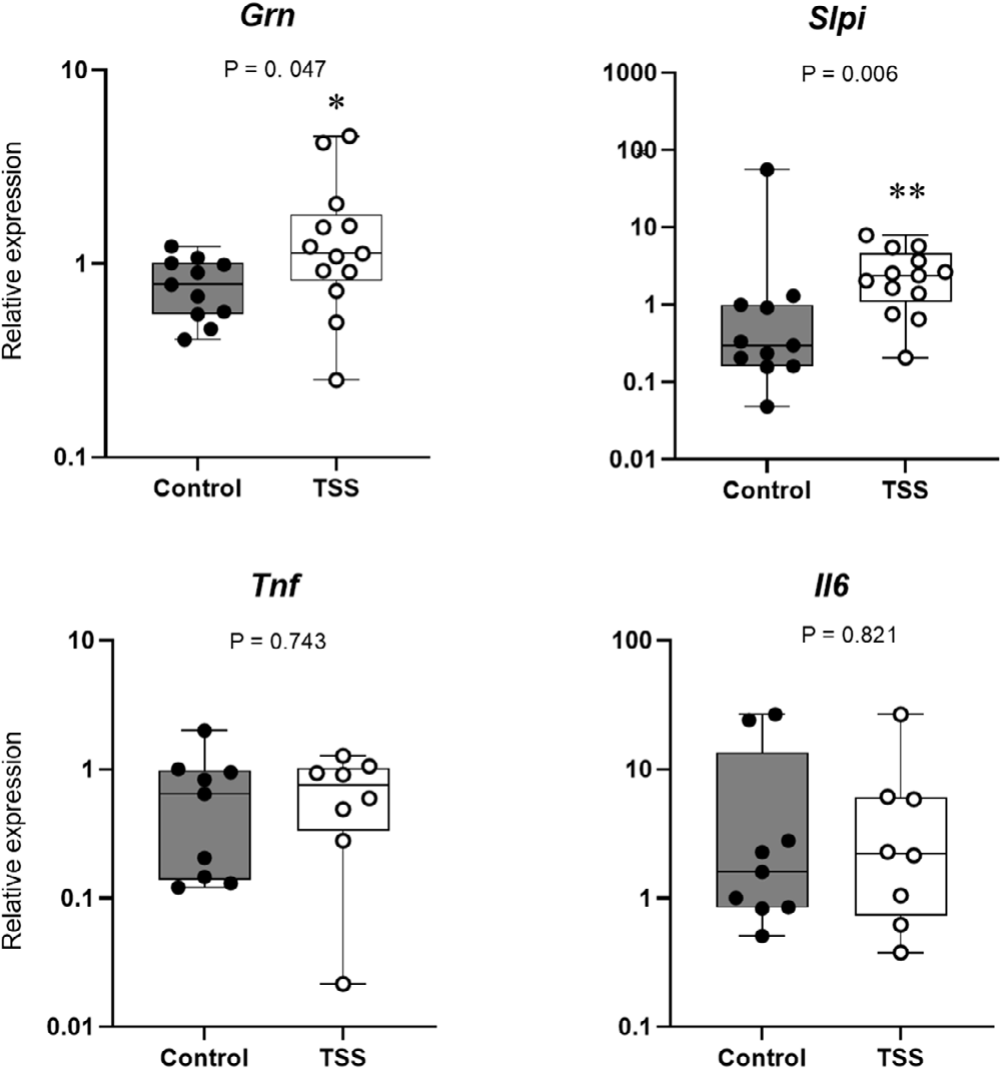
Expression of molecules related to pregnancy homeostasis. The progesterone‐responsive genes and inflammatory cytokines mRNA in corpus uteri were quantified using real‐time PCR. Each value represents the median (25th and 75th percentiles, *n* = 8–13). Statistical significance was determined by Mann‐Whitney test. TSS group increased *Grn* and *Slpi* expression in mifepristone induced pregnancy abnormal model (*p* = 0.047 and 0.006 respectively).

The progesterone‐responsive genes and inflammatory cytokines mRNA of the corpus uteri was examined in mifepristone induced pregnancy abnormal model (Figure 4). We investigated the anti‐inflammatory proteins SLPI and PGRN involved in the mechanism of cervical maturation. 1% TSS group increased PGRN and SLPI expression in mifepristone induced pregnancy abnormal model (*p* = 0.047 and 0.006 respectively, Figure 4). KC, TNF‐α and IL‐6 mRNA showed no change compared to the control group and the 1%TSS group.

## 4 Discussion

In this study, the weaning of progesterone effects by mifepristone administration led to the activation of inflammatory cytokines in the mouse. TSS was demonstrated to have a suppressing effect on the process that led to the induction of inflammatory cytokines, which are produced as a reaction to the weaning of progesterone to the change from a pregnant state to a postpartum state. Furthermore, TSS induced increased the mRNA expression of SLPI and PGRN, which are the molecules with anti‐inflammatory property. These observations are consistent with the possibility that TSS modulates downstream molecules known to be responsive to progesterone. This hypothesis is supported by multiple studies that have demonstrated pharmacological effects of TSS that have been associated with progesterone‐related biological responses. TSS stimulates the secretion of progesterone from corpus luteum and the herbal ingredients, Holen, Peony root, Alisma rhizome or Japanese angelica root contained in TSS is associated with the effect [11, 12]. Moreover, TSS alleviated implantation failure caused by mifepristone in the mouse model, which was associated with restored production of the LIF derived from the endometrial gland [4].

It is well established that the onset of both normal and abnormal labor is preceded by a functional withdrawal of progesterone, which subsequently triggers a shift toward a pro‐inflammatory intrauterine environment [13]. This transition is characterized by increased expression of inflammatory mediators that promote the activation and local recruitment of neutrophils and macrophages, including IL‐6, CXCL1/KC, and MCP‐1 [14, 15, 16]. In consistent with these findings of previous studies, the present study confirmed that mifepristone administration, an antagonist of progesterone receptor signaling, induces a marked increase in the cytokines level associated with neutrophil and macrophage activation, such as IL‐6, KC, and MCP‐1. Mifepristone administration was associated with increased serum G‐CSF levels, consistent with previous reports demonstrating that G‐CSF increases toward the onset of labor. In the present study, TSS administration further enhanced this mifepristone‐induced elevation. At first glance, this finding appears to contradict the established pregnancy‐maintaining effects of TSS. However, G‐CSF is a pleiotropic cytokine with multifaceted roles in pregnancy. In addition to its association with labor, G‐CSF exhibits immunomodulatory properties similar to progesterone, including the induction of regulatory T cells, suppression of Th1‐type cytokines, and enhancement of IL‐10 production [17].

These findings suggest that TSS‐induced upregulation of G‐CSF may represent a compensatory mechanism that counteracts progesterone withdrawal by promoting immune tolerance. This pathway may contribute, at least in part, to the pregnancy‐supportive effects of TSS.

Furthermore, these results are consistent with recent evidence that TSS and its major components, ferulic acid, suppress the inflammatory cytokine production in peritoneal macrophages. Notably, Takeuchi et al. reported that TSS reduced IL‐8 and VEGF in human peritoneal macrophages and endometriotic stromal cell [18]. This research supports the potential anti‐inflammatory and immunomodulatory effects of TSS.

In the cytokine array analysis of the corpus uteri, TSS significantly reduced IL‐6 protein levels. IL‐6 has context‐dependent functions during pregnancy. At physiological concentrations, particularly at the maternal‐fetal interface during early pregnancy, IL‐6 contributes to implantation, trophoblast function, and the establishment of an appropriate local immune environment. In contrast, excessive or dysregulated IL‐6 production is associated with pathological inflammation, pregnancy complications, and the initiation of parturition [19]. IL‐6 promotes cervical ripening, prostaglandin synthesis, and uterine contractility during the transition to labor [19]. Therefore, the present findings should not be interpreted as indicating that generalized inhibition of IL‐6 is beneficial throughout pregnancy. Rather, in this late‐gestation model of mifepristone‐induced functional progesterone withdrawal, TSS may attenuate an excessive pro‐inflammatory response in the uterine corpus and thereby help preserve local uterine homeostasis.

Taken together, these findings suggest that TSS may mitigate inflammatory responses arising from progesterone withdrawal by complementing endogenous progesterone‐mediated immune regulation. This mechanism may underlie the empirical clinical use of TSS to support gestational tissues stabilization and pregnancy maintenance. Notably, the changes in cytokine and chemokine profiles induced by TSS administration were not consistent between peripheral blood and local tissues, such as the uterine corpus and placenta. Specifically, TSS reduced IL‐6 protein levels in the uterus, whereas alterations in circulating cytokines were primarily observed for G‐CSF. Although the underlying mechanisms remain unclear, these findings suggest that TSS may exert differential effects on local uterine inflammation and systemic immune responses following mifepristone administration. In addition, differences in the kinetics of cytokine production and their release into the circulation may contribute to this discrepancy.

Our previous study has demonstrated that SLPI and PGRN are essential regulators of cervical remodeling and uterine immune homeostasis during pregnancy [10]. SLPI contributes to the regulation of local inflammatory responses and the preservation of mucosal integrity, thereby playing a central role in maintaining the structural stability of the fetal membranes [20]. Similarly, PGRN is recognized for its anti‐inflammatory and tissue‐protective properties [21]. PGRN possesses potent anti‐inflammatory effects and may complementarily suppress the heightened inflammatory response associated with progesterone deficiency by inhibiting TNFα signaling and controlling the inflammatory phenotype of macrophages [22]. Importantly, an in vitro study using Ishikawa cells demonstrated that progesterone enhances SLPI expression [23], which supports the concept that SLPI is a downstream effector of progesterone‐mediated uterine quiescence. Against this background, it is particularly noteworthy that the present study found TSS administration to increase SLPI and PGRN mRNA expression in the pregnant uterus. Since mifepristone induces functional progesterone withdrawal and promotes the production inflammatory mediators, an increase in SLPI and PGRN by TSS, which suggests that TSS may attenuate inflammatory responses associated with functional progesterone withdrawal. This interpretation is consistent with previous reports that TSS has exhibited immunomodulatory and anti‐inflammatory properties in various experimental settings.

Taken together, the present results confirmed that TSS mitigates mifepristone‐induced uterine inflammation bypromoting protective anti‐inflammatory pathways involving SLPI and PGRN　, and which contributes to pregnancy maintenance under conditions that progesterone signaling is impaired.

This study has some limitations as follows. To ensure precise dosing, intragastric administration would have been preferable. However, such procedures in pregnant mice may induce stress and increase the risk of miscarriage or fetal loss. Therefore, TSS was administered via ad libitum feeding in this study. No significant differences in maternal body weight were observed between groups, suggesting comparable food intake. However, individual variation in TSS consumption cannot be excluded and may have contributed to variability in the results.

Although TSS administration suppressed IL‐6 production and increased SLPI and PGRN mRNA expression in uterine tissue, the specific cellular sources of these factors and the target cells affected by TSS were not identified. Future studies using spatially resolved approaches will be necessary to clarify the cellular and tissue‐specific mechanisms underlying these effects.

Although SLPI and PGRN were evaluated only at the mRNA level in the present study, previous studies have reported an association between their mRNA and protein expression in endometrial or other biological tissues [24, 25]. Previous human studies have demonstrated a close association between mRNA expression in cervical epithelial cells and protein levels in cervical mucus during labor [26, 27] Nevertheless, these findings cannot substitute for direct protein measurements in our experimental model. Therefore, the biological effects of TSS on SLPI and PGRN protein expression should be interpreted with caution. Future studies incorporating Western blotting, immunohistochemistry, or other protein‐level analyses will be necessary to validate these findings.

In Japan, TSS has long been used for the management of pregnancy‐related complications. Clinically, it is particularly administered to prevent the failure of pregnancy maintenance, including recurrent miscarriage and threatened preterm labor [28, 29]. The findings of the present study suggest that TSS may be associated with progesterone‐responsive anti‐inflammatory pathways, potentially contributing to the suppression of inflammatory mediators as well as the enhanced expression of anti‐inflammatory peptides such as SLPI and PGRN. These immunomodulatory actions could underlie the well‐recognized therapeutic effects of TSS in pregnancy maintenance. Furthermore, our past work revealed that TSS suppresses miscarriage triggered by cytokine storms following aberrant activation of iNKT cells [3], which is emphasis on the of TSS ability to regulate maternal immune tolerance during pregnancy. Distinct from the previously established mechanisms, the present study may propose a novel pharmacological pathway by which TSS supports pregnancy. This is achieved through the regulation of downstream molecules that are known to respond to progesterone and the promotion of an anti‐inflammatory intrauterine environment. This mechanism could represent an additional layer of TSS action in the prevention of pregnancy complications.

## Ethics Statement

This study was approved by the Institutional Animal Care and Use Committee of the University of Tokyo (Registration No. Med‐P18‐092).

## Conflicts of Interest

Y Tokita was an employee of Tsumura & Co. For the other authors, no potential conflict of interest relevant to this article was reported.

## Data Availability

The data that support the findings of this study are available from the corresponding author upon reasonable request.
